# Effect of emergence time on growth and fecundity of *Rapistrum rugosum* and *Brassica tournefortii* in the northern region of Australia

**DOI:** 10.1038/s41598-020-72582-7

**Published:** 2020-09-29

**Authors:** Ahmadreza Mobli, Sudheesh Manalil, Asad Muhammad Khan, Prashant Jha, Bhagirath Singh Chauhan

**Affiliations:** 1 Department of Agrotechnology, Ferdowsi University of Mashhad, Mashhad, 9177948974 Iran; 2 The Centre for Crop Science, Queensland Alliance for Agriculture and Food Innovation (QAAFI), The University of Queensland, Gatton, QLD 4343 Australia; 3 Amrita School of Agricultural Sciences, Amrita Vishwa Vidyapeetham, Coimbatore, 641112 India; 4 Department of Agronomy, Iowa State University, Ames, IA 50011 USA; 5 School of Agriculture and Food Sciences (SAFS), The University of Queensland, Gatton, QLD 4343 Australia

**Keywords:** Ecology, Plant sciences

## Abstract

Weeds from Brassicaceae family are a major threat in many crops including canola, chickpea, cotton and wheat. *Rapistrum rugosum* (L) All. and *Brassica tournefortii* Gouan. are two troublesome weeds in the northern region of Australia. In order to examine their phenology of these weeds, a pot study was conducted in 2018 at the Research Farm of the University of Queensland, Gatton campus with two populations sourced from high (Gatton) and medium (St George) rainfall areas of the northern grain region of Australia. Planting was carried out monthly from April to September, and the growth, flowering and seed production were evaluated. Maximum growth and seed production were observed in weeds planted in April, compared to other planting dates. Biomass of *R. rugosum* and *B. tournefortii* was reduced by 85% and 78%, respectively, as a result of the delay in planting from April to July. *R. rugosum* and *B. tournefortii* produced more than 13,000 and 3500 seeds plant^−1^, respectively, when planted in April and seed production was reduced by > 84% and > 76% when planted in July. No significant differences were observed between populations of both weeds for plant height, number of leaves and biomass, however, the medium rainfall population of *R. rugosum* produced more seeds than the high rainfall population when planted in April. The results of this study suggest that, although *R. rugosum* and *B. tournefortii* were able to emerge in a wider time frame, the growth and seed production were greatest when both weeds were planted in April and there was concomitant reduction in growth attributes when planted in the subsequent months, indicating that management of these weeds early in the cropping season is a prerequisite to population reduction and the mitigation of crop yield losses.

## Introduction

Weeds from the Brassicaceae family are a threat to many crops including wheat (*Triticum aestivum* L.), barley (*Hordeum vulgare* L.), soybean [*Glycine max* (L.) Merr.], cotton (*Gossypium hirsutum* L.), canola (*Brassica napus* L.), chickpea (*Cicer arietinum* L.) and lentil (*Lens culinaris* Medik.)^1–5^. *Rapistrum rugosum* (L.) All. (turnip weed) and *Brassica tournefortii* Gouan. (African mustard) are two troublesome weeds from this family in the northern region of Australia^6,7^. *R. rugosum* and *B. tournefortii*, respectively, are ranked 5th and 6th in terms of infestation of crop regions of Australia, and resulted in AU$ 10.6 and 4.9 m revenue loss per annum, respectively^8^. Both these weeds possess many biological attributes which include their ability to emerge and grow in a wide range of salinity, moisture and temperature conditions^5,9,10^. In addition, these weeds can produce a substantial number of seeds: a single plant of *R. rugosum* and *B. tournefortii* could produce > 77,000 and 9000 seeds plant^−1^, respectively^9,10^ and their protracted germination due to seed dormancy are vital factors leading to their invasive success in many infested areas^9,11,12^. In addition, most of the weeds from this family exhibit allelopathic effects on many other plants^13,14^. Resistance to ALS-inhibitor herbicides has been reported in both weeds^15^. These features have made both weeds a challenging issue for a majority of farmers across Australia.

*Rapistrum rugosum* and *B. tournefortii* can cause significant yield losses in many crops*.* In chickpea, at densities as low as of 10 plants m^−2^ of *R. rugosum,* the yield was reduced > 40%^3^. Manalil and Chauhan^16^ also reported that the presence of 18–24 plants m^−2^ reduced wheat yield by 50%; but a density of 1 plant m^−2^ of *B. tournefortii* only reduced it by 0.35% the wheat yield^17^.

In Australia, seed dormancy levels vary greatly (12–90%) in newly dispersed seeds of *B. tournefortii* biotypes^18^, which contributes to aestivation in hot summer conditions^19^. Likewise, impermeable seed coat results in induced dormancy with lower germination in Australian biotypes of *R. rugosum*^7,10^. Periodic seed germination due to profuse seed production and seed dormancy, as well as the innate ability of these weeds to germinate in a broad range of environmental conditions all contribute to the invasive success of these plants. Weed seeds are able to germinate under a broad range of conditions, and their growth, flowering and seed production can vary according to time of emergence and the environments that these weeds are exposed to during their growth phases^20,21^.

Weed emergence time in relation to crop emergence, interference duration and weed abundance are three factors leading to significant crop yield losses^20–22^. Among these three major factors, weed emergence time is the most significant^23–25^. Potential for peak population rates occur when weeds emerge at an opportune time and are met with favorable environmental conditions of temperature, photoperiod and water availability^26,27^. Hence, study on the emergence time of weeds is important to evaluate population potentials and may provide primary input for decision support systems (DSSs) and the implementation of efficient and viable management strategies^28,29^.

In many studies, it has been reported that the emergence, vegetative growth, seed production and phenological behavior of different weed populations could vary according to environmental conditions^30,31^. This study was conducted to evaluate the effect of weed emergence time on growth, flowering and seed production of two populations of *R. rugosum* and *B. tournefortii* from high and medium rainfall areas.

## Materials and methods

### Seed description

Two populations of *R. rugosum* and *B. tournefortii* were collected in 2017 from Gatton (27.56° S 152.28° E) and St. George (37.96° N 113.56° E), located in the northern region of Queensland, Australia with mean long term rainfall of 770 and 500 mm, respectively. As there was physical seed dormancy for *R. rugosum*, seeds were threshed gently by hand to remove the pod and the recovered naked seeds were used for the study^7^. *B. tournefortii* seeds germinated over 70% throughout the study.

### Experiment details and data collection

**Figure 1:**
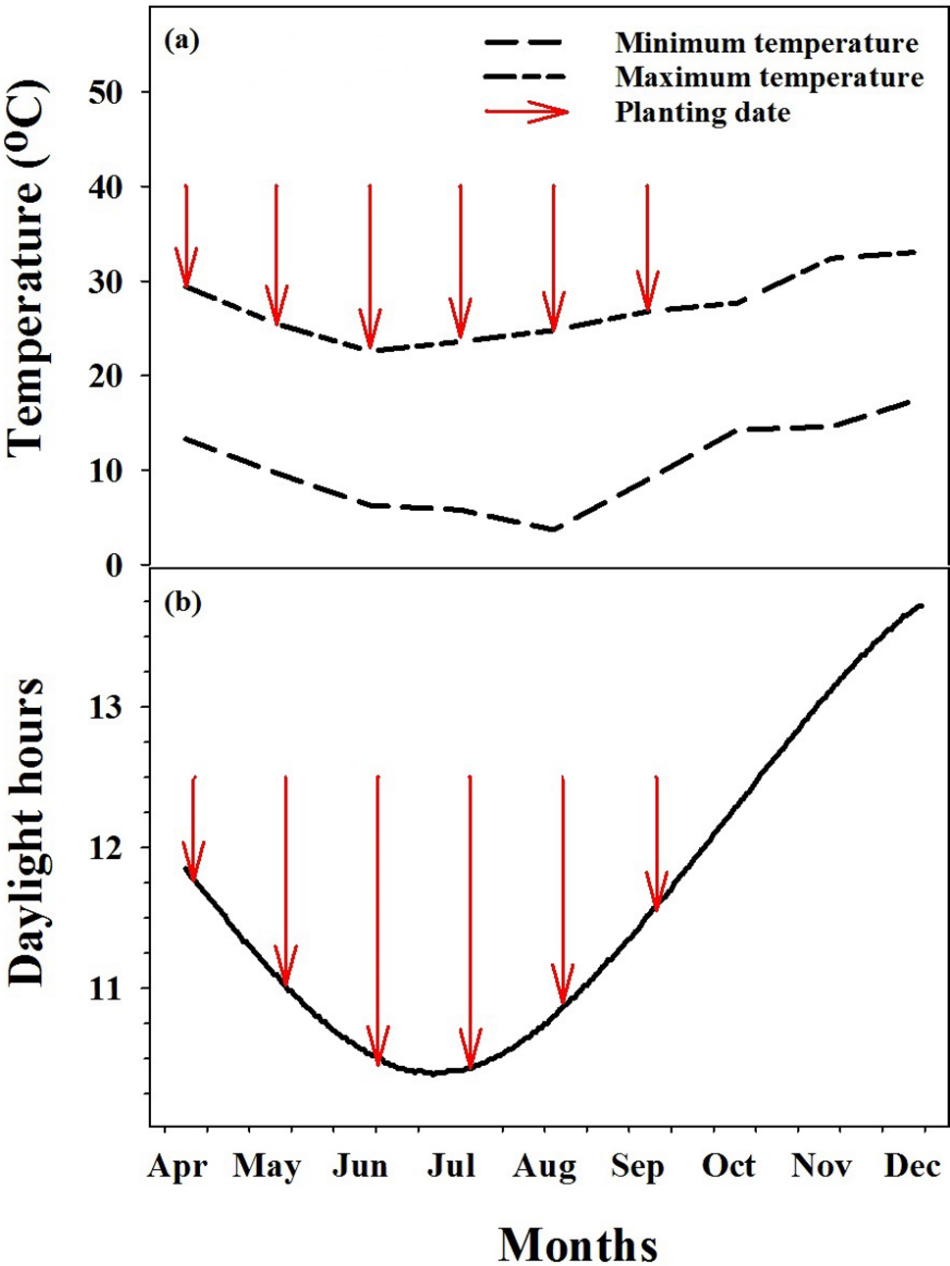
The minimum and maximum temperatures (**a**) and daylight hours (**b**) of Gatton in 2018. The red arrows showed the planting date. The data were adopted from Australian government bureau of meteorology website, available at https://www.bom.gov.au/.

The pot study was conducted at the research farm of the University of Queensland (27.53° S 152.25° E), to assess the effect of planting date of *R. rugosum* and *S. tournefortii* with respect to day length and temperature under field conditions on two populations of each weed in 2018. The minimum and maximum temperatures and daylight hours of the research site are represented in Fig. 1. Plastic pots (35 cm diameter and 40 cm height) filled with field soil with an organic matter of 2.7%, nitrogen of 33 mg kg^−1^, phosphorus of 215 mg kg^−1^ and potash of 412 mg kg^−1^ and pH of 7.2 was used in the experiments. Seeds were sown on the 4th day of every month from April to September (six planting dates) in plastic trays (25 cm × 35 cm) with an emerged seedling at the four-leaf stage transplanted into a pot. Drip irrigation was provided (3 L d^−1^) at two-day intervals. The research area was maintained through periodic hand-weeding. In order to monitor the effect of planting date on the growth of both weeds over time, plant height and number of leaves plant^−1^ were recorded every 2 weeks until plant maturity. Plant height was measured by a metal ruler perpendicularly from the soil surface to the tip of the uppermost leaf. For each planting time, the number of days required for 50% of flowering was considered as a time to flowering. Plants from each planting date were harvested at seed maturity. At harvest, the seeds were thrashed from each plant, and the number of seeds was computed by dividing the 500 seeds weight to total seeds weight. Biomass was taken by cutting the plant at the soil surface and was then placed in an oven set at 70 °C for 96 h. The number of seeds per plant was also recorded.

### Statistical analysis

The experiment was conducted in a complete randomized block design with two factors (factorial arrangement of weed populations × planting dates) for each weed species, with eight replications (48 plants for each population). The statistical analysis was performed by SAS software (version 9), and figures were drawn using Sigmaplot (version 14). Before analysis (ANOVA), the data were tested and satisfied with the normality (Shapiro–Wilk test) and homogeneity (Breusch–Pagan), independency (Durbin-Watson) assumptions, and Student’s t-test was showed the mean error was not significantly different from zero. Except for seed production data of *R. rugosum*, all data were pooled over the populations as no significant differences were observed. The means of plant height and number of leaves were compared with the standard error of means over time. The effect of planting date on growth period, flowering, biomass and seed production was evaluated by least significant difference test at a probability level of 0.05.

## Results

### Effect of planting date on growth period

**Figure 2:**
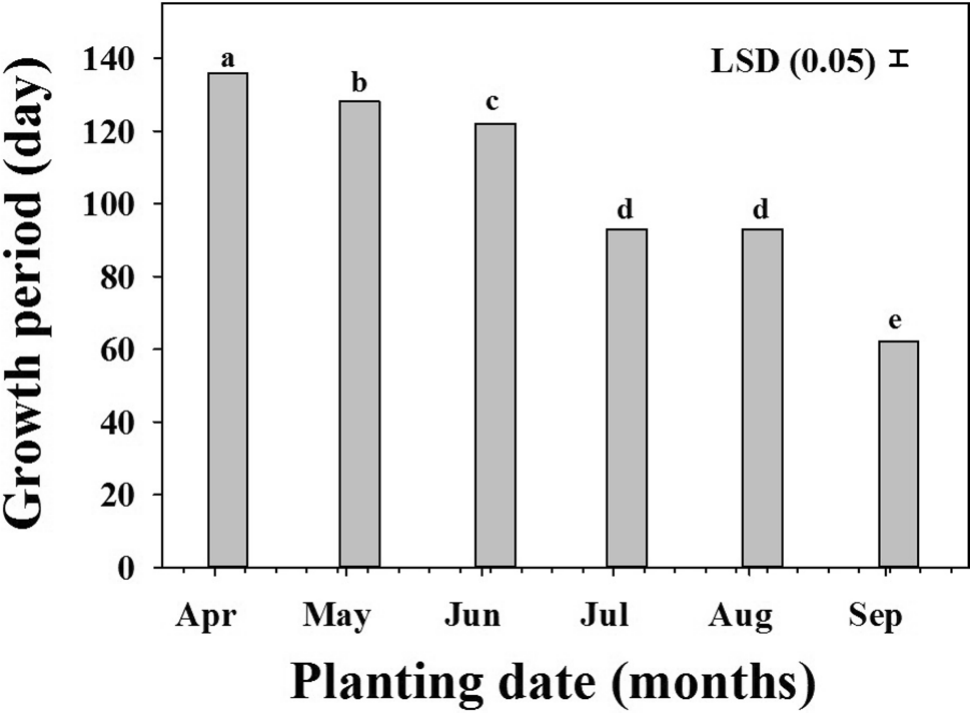
The effect of planting date on *Rapistrum rugosum* and *Brassica tournefortii* growth period. Data were pooled over the populations and species. Vertical bars are least significant difference (LSD) values at the 5% level of probability.

The plant growth period (planting to maturity) in both species was significantly reduced (*P* < 0.001) in response to temperature and daylength period (Fig. 2). The growth period of both weeds when planted in April was of 136 days. When planted in May, June, July and August, the growth period was reduced to 128, 122, 93 and 93 days, respectively. The lowest plant growth period was observed when the populations were planted in the September (62 days).

### Effect of planting date on plant height

**Figure 3:**
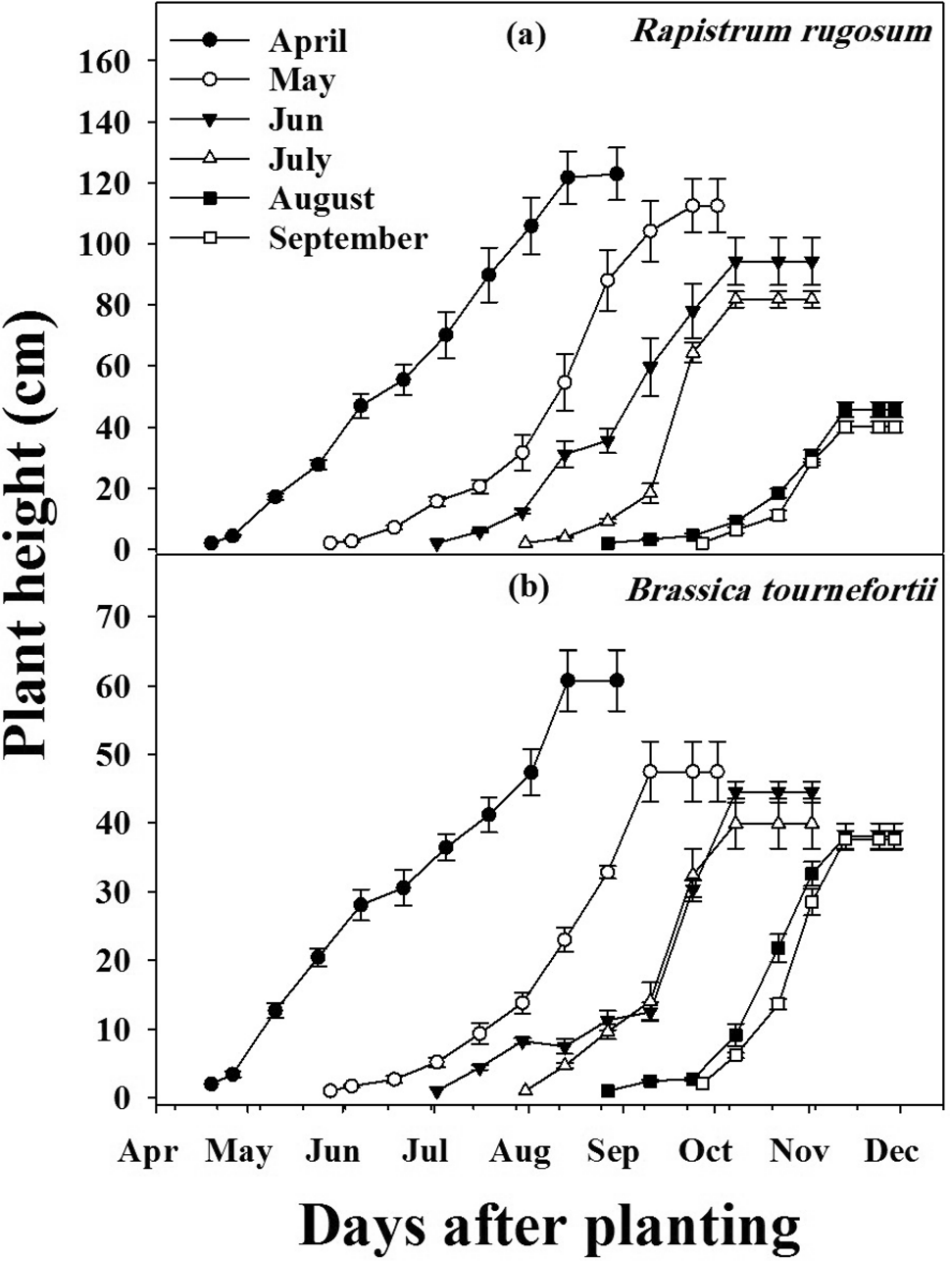
The effect of planting date on *Rapistrum rugosum* (**a**), *Brassica tournefortii* (**b**) height. Data were pooled over the populations. Vertical bars show standard error.

The planting date had a significant effect on the height of both weeds (Fig. 3a,b). Plants sown in April grew taller in comparison with other planting dates. No significant differences were observed between populations of both weed species (*P* < 0.001). A reduction in plant height was observed when planting proceeded from April to September. The maximum reduction in plant height (> 77%) was observed with August planting for both populations of *R. rugosum*. No difference in plant height was observed between planting in August and September. A similar trend was observed for *B. tournefortii*, the height was reduced by > 34% when planted in July in comparison to the planting date in April, and no significant difference was observed between planting dates from July to September.

### Effect of planting date on number of leaves plant^−1^

**Figure 4:**
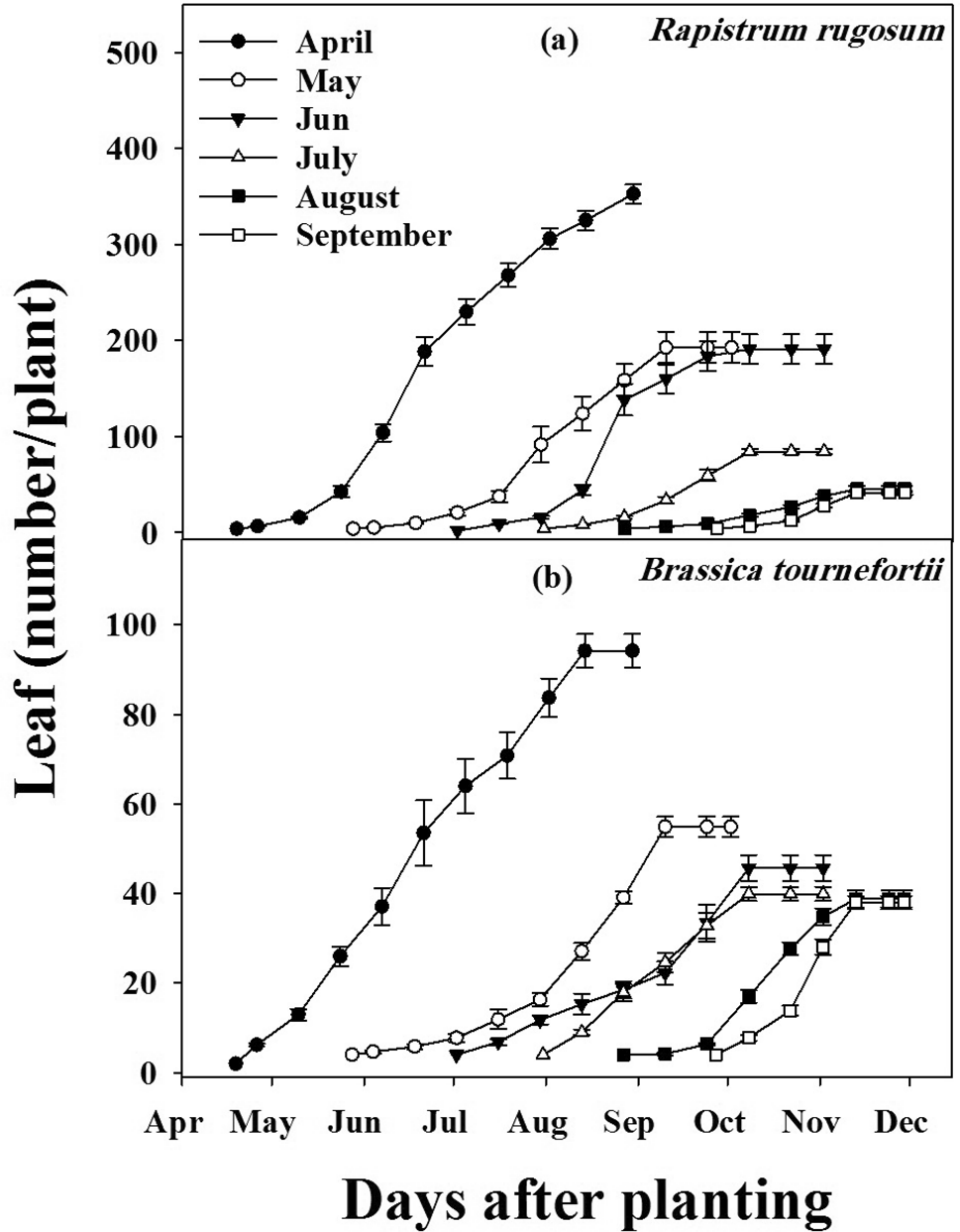
The effect of planting date on *Rapistrum rugosum* (**a**), *Brassica tournefortii* (**b**) number of leaves plant^−1^. Data was pooled over the populations. Vertical bars show standard error.

Similar to plant height, maximum leaves were produced from April planting of *R. rugosum* and *B. tournefortii* (Fig. 4a,b), and no significant difference was observed between populations (*P* < 0.001) A reduction in the number of leaves was observed when planting proceeded further from April to September. Delays in planting date from April to June and July in *R. rugosum* resulted in 56% and 82% reductions in the number of leaves plant^−1^, respectively. Similarly, there was a reduction in the number of leaves plant^−1^ of *B. tournefortii* by 42, 52 and 58% with delayed planting in May, June and July, respectively. Planting from July to September had no significant effect on the number of leaves plant^−1^ for both weeds.

### Effect of planting dates on flowering

**Figure 5:**
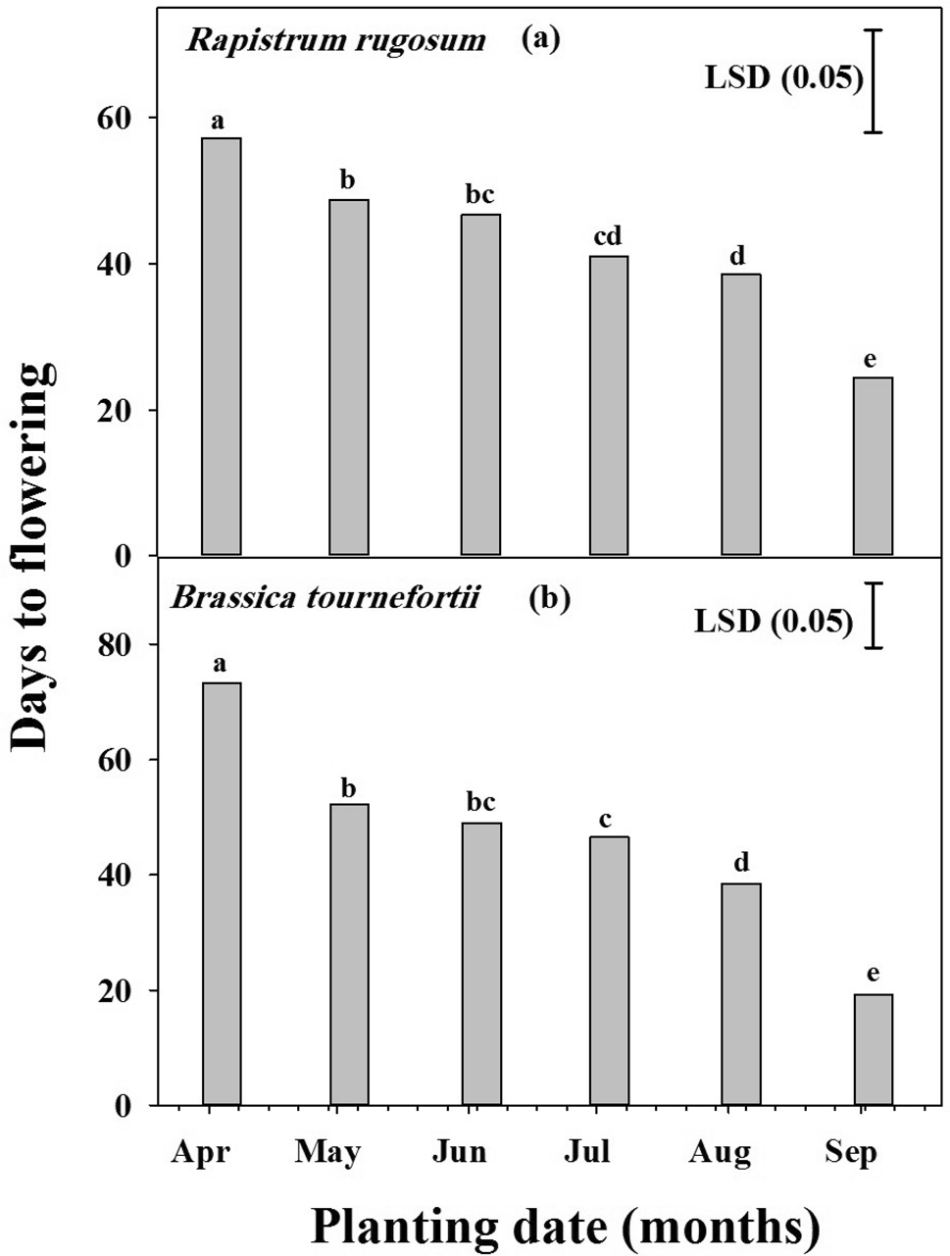
The effect of planting date on *Rapistrum rugosum* and *Brassica tournefortii* flowering. Data were pooled over the populations. Vertical bars are least significant difference (LSD) values at the 5% level of probability.

The days needed to flowering in both species were significantly reduced (P < 0.001) in response to temperature and daylight period (Fig. 5a,b). No significant difference was observed between populations of both weeds (*P* < 0.001). The maximum days needed to flowering was observed for April planting for both *R. rugosum* (57 days) and *B. tournefortii* (73 days). In *R. rugosum*, the delay in planting date from April to May, June, July and August resulted in 15, 18, 28 and 33% reduction in days needed to flowering, respectively. Likewise in *B. tournefortii*, the days needed to flowering was reduced by 29, 33, 37 and 74% with delayed planting in May, June, July and August, respectively. Minimum days needed to flowering was observed for September planting for both *R. rugosum* (24 days) and *B. tournefortii* (19 days) and duration to flowering was reduced by 57 and 74%, respectively.

### Effect of planting dates on biomass

**Figure 6:**
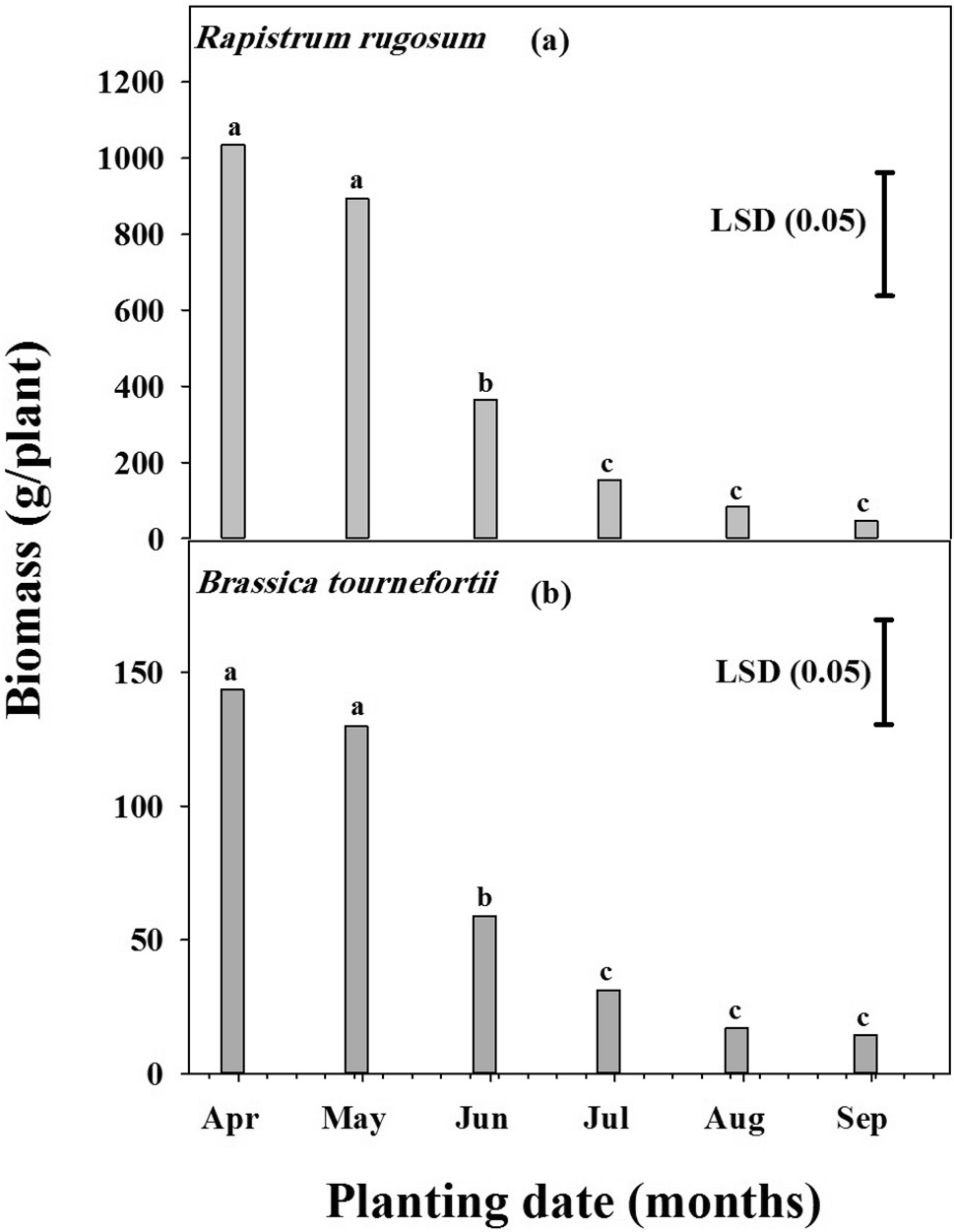
The effect of planting date on *Rapistrum rugosum* and *Brassica tournefortii* biomass. Data were pooled over the populations. Vertical bars are least significant difference (LSD) values at the 5% level of probability.

No significant difference (*P* < 0.001) was observed between biomass of the two populations of both weeds (Fig. 6a,b), and neither was observed in biomass between planting in April and May. However, planting in June and July resulted in a reduction in biomass of *R. rugosum* by 65 and 85%, respectively. Similarly, the biomass of *B. tournefortii* was reduced by 59 and 78% for June and July, respectively, in comparison to that recorded for April. Delay in the planting date from July to September had no significant effect on biomass for both weeds.

### Effect of planting date on seed production

**Figure 7:**
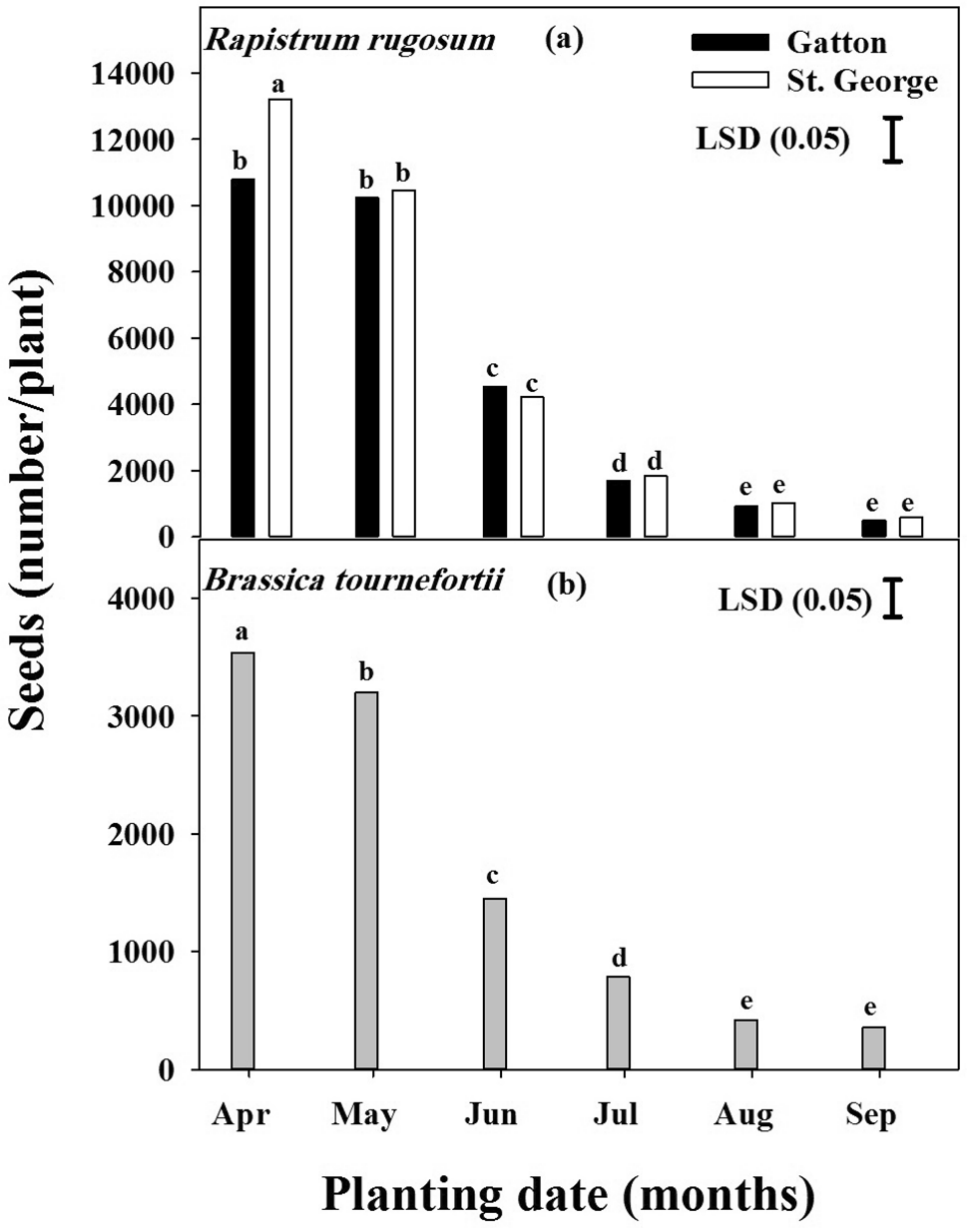
The effect of planting date on *Rapistrum rugosum* and *Brassica tournefortii* seed production. Data of *Brassica tournefortii* seed production was pooled over the populations. Vertical bars are least significant difference (LSD) values at the 5% level of probability.

For seed production of *R. rugosum*, an interaction effect was observed for planting date and population when it was planted in April (*P* < 0.001) (Fig. 7a). The highest seed production was observed for April planting for both St. George (> 13,200 seeds plant^−1^) and Gatton populations (> 10,700 seeds plant^−1^). Seed production in both species was reduced with delayed planting. Compared to planting in April, planting in June, July and August resulted in a reduction of 57, 84 and 91%, respectively, for the Gatton population, and likewise, seed production of the St. George population was reduced by 68, 86 and 92%, respectively. In both populations, no significant difference was observed in seed production with August and September planting. In both populations, > 90% reduction in seed production was observed for the September planted cohorts (500 seeds plant^−1^) compared with the April planted cohorts.

Planting date had a significant effect (*P* < 0.001) on seed production of both populations of *B. tournefortii* and no significant difference was observed between populations (Fig. 7b). Seed production was highest when this weed was planted in April, (> 3,500 seeds plant^−1^). When *B. tournefortii* was planted in June, July and August, there was a reduction in seed production of 59, 76 and 88%, respectively, in comparison with planting in April. No significant difference was observed in seeds produced between the planting dates in August and September.

## Discussion

Environmental conditions often correlate with growth, competitiveness, flowering and seed production in weeds^11,24,25^. When planted in April, both weeds grew taller and produced more leaves, biomass and seeds in comparison with other planting dates. In April, monthly temperature and the daylight hours were at a decreasing phase (autumn season) and these plants flowered in the mid-month of June with an average temperature of 14 °C and a day length of 10:30 h. Our observations indicate that both weeds are ideal competitors in winter growing conditions. Flowering was induced with considerable shortening of the vegetative phase due to enhancement of daylight and temperature. Rapid flowering or shortening of the vegetative phase with planting in July, August and September is correlated with long-day and high-temperature conditions. Although this study has not specifically explored the classification of these weeds based on photoperiod and temperature, these findings indicate both weeds are phenologically long-day plants^32,33^.

A strong relationships between temperature and photoperiod with the phenological stage of some species from the Brassicaceae family have been reported, with these plants adapting to complete their life cycle in accordance with increased daylight hours and temperature^32,33^. Although seed emergence depends upon minimum temperature and light requirements; a hard seed coat and, a rich seed bank often cause seeds to germinate in several flushes^34,35^.

In the present study, the plant growth period and duration to flowering (vegetative growth) in both weeds were reduced with delayed planting date. A sharp reduction in plant height, number of leaves and biomass were observed accordingly. Delay in planting date resulted in both species experiencing warmer and longer days and consequently growing for a shorter period than those planted in April. The decrease in plant height and number of leaves of both species could be attributed to temperature, photoperiod and plant growth duration. A similar response to temperature and photoperiod was also observed in *Sinapis arvensis* L^36^. Many studies have shown that temperature and photoperiod are the main factors that alter phenological stages^37,38^ and, consequently, reduce the growth period of the plant.

Weed phenology is a major factor in weed competitiveness^36^. The ability to estimate the competitiveness of weeds is an undeniable part of integrated weed management^39,40^. The results of this study showed that the biomass of *R. rugosum* and *B. tournefortii* were reduced by 85 and 78%, respectively, as a result of planting in July (90 days of delay compared to planting in April). It could be concluded that any practice, such as application of pre-emergence herbicides, in the early-growth stages can be very useful for control of these two weeds. Such practice may minimize subsequent weed control costs, as any emerged weeds (under lack of early weed control) can grow vigorously and demand more energy and inputs towards their control. Studies also show that the late emergence of *Ambrosia artemisiifolia* L., *Avena fatua* L. and *Amaranthus rudis* J. D. Sauer resulted in reduced competitiveness^41–43^. Interaction of emergence time and competitiveness of these two weeds was not evaluated for a specific study. In future studies, the interactions of emergence time and crop competition should be considered to better understand weed competitiveness over the growth period. Both weeds could germinate and grow throughout the period (April–September) indicating that it would be possible to prompt their emergence through irrigation. Subsequent control through light tillage would serve to deplete the seed bank as emerged weeds are smaller and less competitive.

In the present study, the response of populations collected from high (Gatton) and medium (St. George) rainfall areas were similar except for seed production. *R. rugosum* from St. George produced more seeds than the Gatton population when planted in April. No significant differences were observed between populations of both weeds in plant height, number of leaves and biomass. In contrast, Spaunhorst et al.^30^ observed different growth and seed production between *Amaranthus palmeri* S. Watson populations in response to different planting dates. Different responses in weed populations to environmental factors can be attributed to genetic diversity and maternal conditions over the course of maturation^44,45^.

*R. rugosum* and *B. tournefortii* produced more than 13,000 and 3500 seeds plant^−1^, respectively, when planted in April. In both weed species, seed production was reduced as a result of a reduction in the growth period and duration to flowering (shorter vegetative growth period) with delays in planting. Some studies showed that a decrease in weed seed production could be correlated with biomass production^46,47^. In the current study, delay in planting date from April to August reduced the number of seeds in *R. rugosum* by 90% and in *B. tournefortii* by 88%. In many studies, it was observed that late-emerged weeds produced a lower amount of seeds than early-emerged ones^43,48,49^. Despite this, late-emerging weeds can interfere with harvest operations and may escape from control methods. In this study, in spite of a sharp decline in the number of seeds plant^−1^, more than 500 and 350 seeds were produced in *R. rugosum* and *B. tournefortii* in September, respectively. It could be concluded that, that, despite the reduction in the number of seeds in late-emerged weeds, the control of these weeds (even in boundary areas) should not be neglected in order to achieve sustainable and long-term weed management. In the current study, the viability and dormancy of produced seeds—which play an important role in the proliferation of weeds—from different planting dates were not evaluated^30,39,50^. Furthermore, it has been reported that the interaction effects of flowering time and growth period length could result in different emergence responses to environmental conditions as observed in the collected seeds of *Ipomoea hederacea* (L.) Jacq. from different planting dates germinated in different temperature regimes^50^.

## Conclusions

*Rapistrum rugosum* and *B. tournefortii* germinated throughout the study period (April–December) and germination was not inhibited under field conditions, though both species are considered predominant weeds of the winter season. Maximum growth and reproductive potential of these weeds were observed when planted in April; these weeds grew taller and produced more leaves, biomass and seeds in comparison with other planting dates. The relations between temperature and photoperiod on the phenological stage showed the tendency to flower by starting to increase daylight hours and temperature, leading to a shorter vegetative phase. The growth and seed production of both weeds were reduced as a consequence of the reduction in the growth period due to rapid flowering when planting was delayed. Control of *R. rugosum* and *B. tournefortii* in early-season can be useful to minimize subsequent weed control costs, as any emerged weeds early in the season can grow vigorously and demand more energy and inputs towards weed control. Although late cohorts of weeds produced a low number of seeds, the control of late-emerged weed plants should not be neglected to achieve sustainable and long-term weed management.
